# Assessing Objective and Verifiable Indicators Associated With Work-Related Stress: Validation of a Structured Checklist for the Assessment and Management of Work-Related Stress

**DOI:** 10.3389/fpsyg.2018.02424

**Published:** 2018-12-04

**Authors:** Claudio Barbaranelli, Valerio Ghezzi, Cristina Di Tecco, Matteo Ronchetti, Roberta Fida, Monica Ghelli, Benedetta Persechino, Sergio Iavicoli

**Affiliations:** ^1^Department of Psychology, Sapienza University of Rome, Rome, Italy; ^2^Department of Occupational and Environmental Medicine, Epidemiology and Hygiene, Italian National Workers Compensation Authority, Rome, Italy; ^3^Norwich Business School, University of East Anglia, Norwich, United Kingdom

**Keywords:** work-related stress, risk assessment, checklist, multi-method approach, formative indicators

## Abstract

Risk assessment represents an essential part of any successful intervention in health and safety at work. The most prominent European methodologies propose multi-method approaches for identifying the risks associated with work-related stress. Nevertheless, the most widely used method is the self-administered questionnaire. By adapting the UK Management Standards approach, the Italian National Workers Compensation Authority (INAIL) developed a checklist for the assessment of objective and verifiable indicators of work-related stress. This checklist is filled in by a steering group composed of homogenous groups of workers. Through a web-platform developed by INAIL, a considerable amount of data over the last 5 years has been collected throughout Italy. The aims of this study are to examine the psychometric properties as well as the practical validity of the checklist in a wide sample of Italian companies. The sample comprised 5,301 homogeneous groups of workers nested within 1,631 organizations. The checklist measures two main areas: (1) the organizational indicators of work-related stress (sentinel events) and (2) four and six factors related respectively to content and context of work. Multilevel and multivariate analyses revealed that the checklist shows adequate factor structure and criterion validity. Results also demonstrate that small companies and the public and healthcare sector show higher risk levels. These results support the use of the checklist as a structured and generalizable tool for assessing and monitoring the risks associated with work-related stress.

## Introduction

Psychosocial risks have been identified among the main emerging risks for health and safety and a key priority for research and policy (Leka et al., 2010, 2015; Iavicoli et al., 2011; EU-OSHA, 2015). These are “those aspects of work design and the organization and management of work, and their social and environmental contexts, which have the potential for causing psychological, social and physical harms” (Cox and Griffiths, 1995, p. 69).

Interest in studying psychosocial factors has grown in particular due to their link with work-related stress (Bongers et al., 1993; Siegrist and Wahrendorf, 2009; Nieuwenhuijsen et al., 2010; Kivimäki et al., 2015). Indeed, a poor psychosocial work environment may result in the emergence of work-related stress, which in turn may affect workers’ health. Accordingly, policy actions, agreements at national or organizational level, approaches and specific tools and methods were developed over time to foster the assessment and management of psychosocial risks into organizations (European Trade Union Congress, 2004, 2007; European Commission, 2011).

Comparative evaluations of the most prominent methods identified that one crucial element linked to their success in improving workers’ health and wellbeing is represented by the availability of instruments for risk assessment embedded within the more general frame of a risk management approach to health and safety (Leka and Cox, 2008; Nielsen et al., 2010; Nielsen and Randall, 2013). Indeed, risk assessment represents a pivotal phase of any successful intervention program in health and safety in order to plan fitting interventions (Eurofound and EU-OSHA, 2014). In light of this, the selection of methods and tools for the assessment is fundamental.

Due to the multi-faceted nature of work-related stress, integrating different data and assessment methods can be useful to obtain a more complete identification of the risks (Regulies, 2012). Nevertheless, self-report questionnaires measuring workers’ perceptions of their working conditions are generally the only tool offered to companies (Nielsen et al., 2010), since they are easily validated and administrable to large numbers of workers in different workplaces. Conversely, the use of instruments such as semi-structured interviews and checklists may be deterred by the major complexity of the procedures for data collection and psychometric validation. Accordingly, most of the available methodologies generally limit their offer to self-report questionnaires, without considering the collection of organizational records as well as of observational information.

The purpose of the current study is to investigate the psychometric characteristics of a checklist developed by the Italian National Workers Compensation Authority (henceforth, INAIL) for the assessment of objective and verifiable indicators associated with work-related stress. While there have been preliminary studies on this tool (e.g., Di Tecco et al., 2015; Ronchetti et al., 2015), the present study represents a first extensive contribution to the investigation of the psychometric characteristics of this instrument, capitalizing on the large data base collected by INAIL during the last 5 years within the frame of INAIL’s methodology for the assessment of psychosocial risk factors. In the following sections, we will briefly describe how this checklist has been developed and preliminarily studied.

### The INAIL’s Checklist for the Assessment of Risks Associated With Work-Related Stress

In 2008, the European policies were implemented into the Italian normative framework for health and safety stating that the employer has a duty to assess all risks for health and safety, including those associated with work-related stress. This led to a flourish of national initiatives and proposals to companies facing this requirement.

The Department of Occupational and Environmental Medicine, Epidemiology and Hygiene of INAIL established a multidisciplinary group, which developed an evidence-based and integrated methodological process for supporting Italian companies in the management of risks associated with work-related stress. This methodological proposal was framed within the Management Standards (MS) approach, developed by the UK Health and Safety Executive (HSE: Cousins et al., 2004; Mackay et al., 2004; Edwards et al., 2008) adapted to the legal requirements of Italy and complemented by other national existing experiences. In adapting this approach to the Italian context, it has been acknowledged the lack of a structured tool for a preliminary assessment of objective and observational data linked to work-related stress (e.g., absences from work, injuries, aspects related to the organization of work, work context, etc.). Indeed, while the HSE’s approach calls for the examination of such information in the risk assessment, it does not offer any specific structured and standardized tool for collecting these data. This absence raised concerns regarding a full applicability of the HSE’s approach (Iavicoli et al., 2009), since the preliminary assessment of such data represents a minimal requirement according to the Italian legal system. To fill this gap, INAIL adopted a structured checklist for collecting and assessing organizational objective records and observational data among the tools for the assessment of organizational risk factors for work-related stress.

The checklist is the result of the adaptation and integration of a tool previously developed by the Italian National Steering Network for the Prevention of Work-related Psychosocial Disorders (henceforth, the “Network”), established in 2007. The “Network” involved representatives of the clinical centers for disorders related to psychosocial risks and experts from several regional units for occupational prevention, with the aim of promoting dialog among experts on local existing experiences, in order to reach a common proposal for the assessment of work-related stress. Starting from a review of the main theoretical models and approaches on psychosocial factors associated with work-related stress, the “Network” developed a checklist as a tool for the assessment of some organizational records and indicators that may be signals of work-related stress. The final checklist (henceforth I-Check, standing for INAIL-Checklist) comprises two parts: the first part regards the assessment of “sentinel events” as indicators of work related stress to identify potential evidence of its presence; the second part regards the assessment of those aspects of work content and work context, which are considered determinants of work-related stress.

The first part of the I-Check includes the most common organizational records of indicators linked to work stress (European Trade Union Congress, 2004) - as sickness absences, work-related injuries, and turnover (see Table 1 for further details) - and generally collected by companies. These have been labeled “sentinel events” since they are considered possible signals of the experience of work-related stress in the organization (Salminen, 2004; Eurofound and EU-OSHA, 2014; Allisey et al., 2016). This part of the checklist comprises only objective data collected from administrative sources.

**Table 1:** Areas and dimensions and the I-Check.

Part 1	Part 2	
	
Sentinel events	Work content factors	Work context factors
Work-related Injuries	Work Environment	Function
Sick leave absences	Task	Role
Absences from work	Workload	Career
Left-over vacation days	Schedule	Autonomy
Job Rotation		Relationships
Turnover		Home/Work Interface
Legal actions/disciplinary sanctions		
Requests for extraordinary visits		
Formal records of employees complaints to the company or to the company’s physician		
Legal applications		


The second part of the I-Check refers to Cox’s framework (1995) on the assessment of the psychosocial hazards. In the 1990s, Tom Cox developed a taxonomy of psychosocial hazards based on a comprehensive literature review (Cox, 1990; Cox and Cox, 1993; Cox and Griffiths, 1995) and summarized *ten* work and organizational, environmental and job characteristics, which may have the potential to cause harm to employees if they are inadequate or insufficient. The same characteristics, if well managed, may instead have a beneficial effect. Following a traditional distinction in Occupational Health Psychology (Hacker et al., 1983; Hacker, 1991) these *ten* psychosocial hazards included in the Cox’s taxonomy may be conceived as relating to two areas: *work content* and *work context* factors. Psychosocial hazards related to the *content* of work are those aspects of the content of the work that may have a potential stressful effect on workers, namely: work environment and work equipment, task design, workload/work-pace and work schedule. Psychosocial hazards related to the work *context* are aspects and characteristics of the work context that may be considered stressful by employees if poor: organizational culture and function, role in organization, career development, decision latitude/control, interpersonal relationships at work and home-work interface. Cox’s taxonomy is recalled in a work commissioned by EU-OSHA (2000) where an updated and detailed review of studies on work-related stress confirmed those psychosocial hazards.

While Cox’s taxonomy identifies work content and work context risk factors, it does not provide a specific set of indicators for measuring them. In the development of the I-Check, thus, a crucial step was the identification of the indicators for each work characteristic (or psychosocial hazard). This has been done using a *bottom up approach* where, starting from the work related stressors’ taxonomy, researchers identified indicators for each work content and work context factor describing most common work conditions and organizational critical situations. These indicators were identified by the “Network” considering also what an organization should be doing to manage each of the ten factors recognized as being important to be managed in order to reduce the hazard of work-related stress. In this process of item generation, all indicators describing most common situations and procedures were included by researchers, irrespective of whether these are similar or correlated aspects and regardless of the type or the size of organization. Then, following suggestions from previous experiences in this field (Cousins et al., 2004; Mackay et al., 2004), a first list of indicators for the hazards factors was then presented and discussed in a series of work groups organized in the context of the “Network” activities, involving main stakeholders and experts in the occupational health and safety field. Following a multidisciplinary approach, the experts involved were different stakeholders as academics, trades union representatives, OSH professionals, researchers, labor inspectors, employers, occupational psychologists and occupational physicians. During the work groups, a drafted checklist including a large number of indicators for each psychosocial hazards was presented to the experts for discussion. The main aim of the work groups was to select and revise indicators so that all aspects of work context and content factors linked to the prevention and management of work-related stress were adequately analyzed. The final checklist included all the indicators describing key aspects linked to each particular hazard. Moreover, in selecting the final set of items, experts paid particular attention to prioritize the clearest and generalisable indicators linked to concrete risk management interventions and preventive actions.

An important consequence of such approach is that indicators of hazard factors are not an effect of these work content and work context hazard factors: they contribute instead at evidencing the potential harm in each specific factor. Accordingly, indicators describing the same psychosocial hazard are not necessarily correlated with each other, and are not conceptually interchangeable, since they were identified to describe most of the possible and different harmful aspects and conditions that are conducive to work-related stress. We believe that these indicators are better conceptualized as “formative” rather than “reflective” measures of the 10 latent dimensions. These dimensions, in fact, represent the different factors of psychosocial hazard *emerging* from those work characteristics (content) and those aspects of a psychosocial work environment (context) having the potential for psychological harm. As documented by a vast literature (for a detailed description see Appendix A in the Supplemental Material) formative (or *causal*) indicators influence the latent variable directly, they are not caused by the latent variable, but they “jointly determine the conceptual and empirical meaning of the construct[s]” (Jarvis et al., 2003, p. 201). We believe this conceptualization is more compatible and suitable to capture the nature of indicators of work content and work context factors.

The I-Check was developed to be filled-in by a steering group for the assessment and management of work-related stress, composed by the employer (or a representative), the organizational Safety and Health professionals and the workers’ representative/s for health. The steering group is bound to collect all the data, information and documents necessary to fill-in the I-Check in order to provide the most realistic and objective description of the work environment aspects measured by the indicators. The involvement of workers – and/or their representatives – in filling in the checklist was considered necessary since they are the best informants on the real conditions and characteristics of their work. Companies must fill-in the I-Check referring to homogenous groups of workers. These are groups of workers that are homogenous in relation to being exposed to common risk factors. The focus of the assessment on homogenous groups has been considered particularly useful in order to orientate more effective interventions since it enables the identification of the common risks features related to both the job and the context shared by workers.

### Preliminary Studies on the I-Check and Aims of the Current Study

Soon after the development of the I-Check, the Veneto Region ASL20 Occupational Prevention, Hygiene, and Safety Service, in collaboration with the University of Verona, began a follow up study on 800 companies adopting the I-Check. This study was aimed to collect feedback on the clarity and full understanding of indicators, completeness in terms of information required and the feasibility in compiling it, using expert feedback. This represented the first analysis of the I-Check characteristics through field-testing. However, there was a need to verify in depth its psychometric characteristics in a larger sample. Thus, INAIL’s team developed a web platform consisting of a web interface where companies have free access to the online tools developed by INAIL - including the I-Check - to carry out the assessment and management of risks associated with work-related stress. This web platform was developed with the aims to support companies with a web interface for using the tools and downloading useful materials, allow the upload of data in order to develop findings’ reports, and create a structured repository where assessment data from companies are constantly collected, with the aim of optimizing and standardizing the tools over time.

Thanks to the development of this web platform, a considerable amount of data has been collected in the last 6 years (over 6,000 completed I-Checks, May 2016). This enabled two further preliminary studies (Di Tecco et al., 2015; Ronchetti et al., 2015). Findings from such studies showed a high level of satisfaction among companies in using the I-Check in the assessment of risks associated with work stress and its substantial complementarity with self-report instruments. In particular, in a sample of 137 organizations Ronchetti et al. (2015) showed that the higher the risks associated to work-related stress obtained through the I-Check, the higher the risk perceived by workers measured with the Management Standards Indicator Tool (MS-IT, Edwards et al., 2008). This represents a first important evidence of the convergent validity of the Checklist with a validated tool (for the Italian validation of the MS-IT see Rondinone et al., 2012). However, due to the sample limitation in these preliminary studies, the psychometric features of the I-Check were not fully addressed. This raised concerns regarding the legitimate use of the I-Check (e.g., Balducci, 2015; Corradini et al., 2016).

With study we aim at investigating:

(1) the psychometric properties of the I-Check, with particular emphasis on the measurement model of the work content and context factors, using a large sample of groups and organizations within a multilevel approach; in particular we hypothesize to find the same 4-factor and 6-factor structure respectively for work content and context factors, both at the level of the homogeneous groups and of the organizations considered.
(2) The relationships among work content and context factors, objectively measured indicators (e.g., absences from work, injuries, sick leave days) and the available organizational variables. In particular we hypothesize to find at the organizational level positive correlations between I-Check work content and context factors, and the total score of sentinel events as well as the “risk balance” indicator (see below). We also hypothesize that organizations classified into different levels of risk, having different size and operating in different economic sectors will show different risk profiles in work content and context factors.

These aims were realized on a large sample of Italian companies using INAIL’s methodology for the assessment and management of risks associated with work-related stress.

## Materials and Methods

### Procedure

As explained in the introduction, in INAIL’s methodology the assessment of risks associated with work-related stress is conducted on homogenous groups of workers (generally depending on the size of company and the organizational complexity). Accordingly, companies are asked to fill-in one I-Check per homogenous group of workers, with the aim of having two or more checklists completed for each company. Thus, homogenous groups nested within the referent companies that used the I-Check constituted the basic statistical units of the present study. The checklists were filled-in by companies’ steering groups that collected data from organizational archival records for each homogeneous group (or for the entire company in the case of fewer than 30 employees) to verify the presence of modifications over the last 3 years in some relevant organizational records associated with work-related stress, namely the sentinel events. Concerning the work content and work context aspects, the steering groups collected information and documentations useful for describing the real work environment aspect as objectively as possible and began a group discussion to answer to the indicators. As noted above, workers representing the homogenous groups and/or their representatives for health are involved in the group discussion as the best informants of the work and contextual aspects. Once completed, data collected through the I-Check were uploaded onto the INAIL’s web platform to enable the reporting of findings.

### Participants

Thanks to the development of INAIL’s web platform, a considerable amount of data has been collected in the last 6 years. Using this dataset as the starting point, homogenous groups to be retained in the final sample were selected according to the following criteria:

(1) Completion of the preliminary assessment up to March 2016;
(2) Exclusion of homogeneous groups where consulting firms filled-in the I-Checks for one or more companies by registering with their own credentials instead of the companies’ ones.

Homogenous groups that did not meet this entire set of eligibility criteria were excluded from the sample for the present study. The initial overall dataset consisted of more than 6,000 homogenous groups corresponding to 2,463 companies. After applying the eligibility criteria discussed above, the final sample consisted of 5,301 homogenous groups nested within 1,631 organizations (average number of homogeneous groups per organization is 3.25, with a SD of 7.04). Most of the companies in the present study were involved in manufacturing (22.75%), professional, scientific, technical activities or activities of extraterritorial organizations and bodies (16.31%) and wholesale, retail trade, accommodation and food service economic sectors (13.98%). The 71.30% of organizations in which homogeneous groups were nested had 50 or less employees, while 5.63% of companies had more than 1,000 employees. Complete sample statistics pertaining to the organizations are provided in Supplementary Table S1.

### Ethics Statement

There are not single human participants revealing personal information during the data collection. Moreover, this study uses desk data collected within the assessment procedure for work-related stress conducted at the organization level. These data are stored within the system developed by INAIL and are accessible only to each single organization as far as the specific report related to that single organization. INAIL made the data base available to the researchers involved in this study once the name of each single organization in the data base has been obscured. This research is based on a secondary analysis of de-identified data provided by INAIL and, for this reason, approval from the ethical committee of the academic institutions involved in this study is not needed.

### Measures

The instrument used for the present study is the I-Check (INAIL, 2013) whose development was described in a previous section. In this paragraph, we present an in depth description of the different parts of this tool. As noted above, the I-Check consists of two main areas, namely: (a) organizational indicators of work-related stress, which are called *sentinel events*; (b) factors related to the content and the context of work, namely *work content factors* (henceforth “*content*”) and *work context factors* (henceforth “*context*”).

#### Area 1

*Sentinel events* (SE1-S10): these consist of 10 indicators representing organizational modifications over the last 3 years in some relevant organizational records associated with work-related stress. These data are calculated by the steering group compiling the I-Check from organizational archival data and records for each homogeneous group (or for the entire company in the case of fewer than 30 employees). Specifically, sentinel events included in the I-Check are: SE1. Work-related injuries; SE2. Sick leave absences; SE3. Absences from work; SE4. Left over vacation days; SE5. Job rotation; SE6. Turnover; SE7. Legal actions/disciplinary sanctions; SE8. Requests for extraordinary visits; SE9. Formal records of employees’ complaints to the company or to the company’s physician; SE10. Legal applications. The indicators from SE1 to SE8 are scored on three ordered categories (decreased, unvaried, increased with respect to the last 3 years). The indicators SE9 and SE10 are dichotomous (if they have been reported or not in the last 3 years) since the data measured by such indicators are considered evidence of an experience of work-related stress. It is noteworthy that all these indicators are derived from objective organizational records and it is possible to refer to the user manual of INAIL’s methodology for the methods of calculations adopted by companies (INAIL, 2013, 2017). Since sentinel events represent single item variables, they were excluded from the analytic procedures for testing the measurement model of the I-Check that will be described in the next pages.

#### Area 2

*Work Content Factors*: these identify some key dimensions related to stress at work associated with the content of a specific job. The *content* dimensions are: (1) Work environment and Work equipment (*Work Environment*) measured by 13 indicators (e.g., ‘Suitable microclimate,’ ‘Noise exposure exceeding the second level of action’); (2) Task planning (*Task*) measured by six indicators (e.g., ‘Frequent interruptions at work’; ‘Clear definition of tasks’); (3) Workload – Work pace (*Workload*) measured by nine indicators (e.g., ‘Job characterized by high repeatability’; ‘Fixed work rate for the execution of the task’); (4) Work schedule (*Schedule*) measured by eight indicators (e.g., ‘Work schedules change frequently’; ‘Presence of shift work’).

*Work context factors*: these represent some core aspects regarding work-related stress pertaining to the context in which a job takes place. The *context* dimensions are: (1) Function and Organizational culture (*Function*) measured by 11 indicators (e.g., ‘Company’s procedures are illustrated to employees’; ‘Presence of meeting between management and employees’), (2) Organizational role (*Role*) measured by four indicators (e.g., ‘Roles are clearly defined’; ‘Employees have multiple overlapping roles,’ (3) Career path (*Career*) measured by three indicators (e.g., ‘Presence of a defined career advancement path’), (4) Autonomy in decision making – Job control (*Autonomy*) measured by five indicators (e.g., ‘Work depends on the activities previously carried out by others’; ‘Presence of strict job monitoring protocols’), (5) Interpersonal relationships (*Relationships*) at work measured by three indicators (e.g., ‘Conflicts with managers and colleagues are properly managed’), (6) Home/Work interface – Home/Work balance (*Home/Work interface*) measured by four indicators (e.g., ‘Opportunity to perform vertical and horizontal part-time work’).

The 36 indicators used for measuring the four *content* factors and the 30 indicators used for the measure of six *context* factors are measured on a dichotomous scale (Yes, No). For further information related to the risk scoring system it is possible to consult the user manual of INAIL’s methodology (INAIL, 2013, 2017).

### Analytic Strategy

As explained in the introduction, *content* and *context* indicators were originally formulated adopting a bottom-up approach. As noted above, this approach is consistent with the conceptualisation of content and context indicators as “formative.” A set of analyses were then aimed at investigating the measurement model of *content* and *context* factors. In this regard, a large body of literature has been written on the different conceptualisation of formative (or *causal*) and reflective (or *effect*) indicators (see among others Diamantopoulos and Siguaw, 2006; Bollen and Davis, 2009; Bollen and Diamantopoulos, 2017). For a conceptual overview about the distinction among formative and reflective indicators, see Appendix A of Supplementary Materials.

**FIGURE 1:**
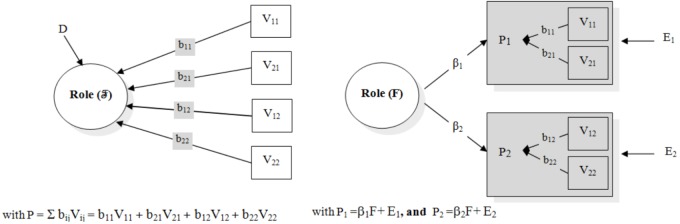
Formal Representation of Treiblmaier et al. (2011) Applied to the Formative Latent Variable “*Role.*” **(Left)** Structural representation the conceptual model for the formative factor Role (F). **(Right)** Structural representation of approximation of the formative factor Role (F) by means of the *reflective factor* Role (F).

Recently, Treiblmaier et al. (2011) proposed a procedure for implementing formative measurement models which seems to overcome the problems related to the specification of these models (see in this regard Cadogan and Lee, 2013; Lee and Cadogan, 2013). This procedure is based on a two-step approach which has the advantages of: (1) producing measurement models that are identifiable *per se*; (2) defining latent variables whose meaning is determined only by their antecedent indicators, and which does not change when they are embedded in a broader structural equation model. In Step 1 of this approach, maximally correlated composites of indicators are identified for each latent variable and optimal scoring weights are developed with a set of canonical correlation analyses. Then, in Step 2, reflective factors are posited considering as indicators the optimally correlated composites weighted as derived by Step 1 of this procedure. A formal representation of formative latent variable implemented via common factor is presented in Figure 1 (with “Role” as an example), while a technical description of Treiblmaier et al. (2011) procedure is provided in the Appendix B of Supplementary Materials.

Since the indicators pertaining to *content* and *context* formative dimensions were ordered categorical variables, we carried out a series of non-linear canonical correlation analyses (one per each ‘formative’ dimension) on the 10 different item pools that defined respectively the four *content* and the six *context* factors. In each analysis, the indicators pertaining to a formative construct were split into two sets by splitting each maximally correlated pairs of items in separate sets (if the number of items were odd, the remaining item was included into the set to which it showed the highest correlation), and optimal weights (i.e., *canonical coefficients*) were identified in order to implement Step 1 of Treiblmaier et al. (2011). These coefficients were then used to weight observed variables forming the two composites in order to derive two item-composites as indicators for each one of the *content* and *context* factors. Since *Career* and *Relationships* were measured only by three indicators, their composite score was posited as a single indicator latent variable (Bollen, 1989) fixing to zero their residual variances at both levels of analysis to avoid convergence problems in further analyses (Heck and Thomas, 2015). In case of negative but not significant residual variances of any composite score at the homogenous group or organizational level, they were fixed to zero and the model was re-estimated after introducing such constraints (on this point, see Heck and Thomas, 2015).

Homogenous groups are nested within organizations. Thus, clustering effects on the item-composites derived from Step 1 of the Treiblmaier et al. (2011) procedure were evaluated by calculating the Intraclass Correlation Coefficient (ICC) and the design effect (*Deff*) index (see Heck and Thomas, 2015). An average ICC > 0.10 (Hox, 2010) and an overall *Deff* around 2 (Muthén and Satorra, 1995) suggest the adoption of a multilevel strategy to test the posited measurement model. In cases such as this, the multilevel confirmatory factor analytic model is particularly suitable for testing the hypothesized formative model. Specifically, a factor solution was tested simultaneously positing 10 correlated latent variables (i.e., four content and six context factors) both at the homogenous group and the organizational level. Model fit was evaluated by: (i) the chi-square test (if not significant, the fit of the model with the observed data is perfect). However, in our case, the null hypothesis of this test may be easily rejected due to the large sample size; (ii) comparative fit index (CFI: Bentler, 1990); (iii) root mean square error of approximation (RMSEA: Steiger, 1990) along with the test of close fit; and (iv) standardized root mean squared residual (SRMR: Hu and Bentler, 1999). Moreover, in order to evaluate potential model misspecifications at each level of analysis, the aforementioned RMSEA and CFI fit indices were evaluated following the *partially saturated model* approach devised by Ryu and West (2009) and Ryu (2014). Both for overall and level-specific model evaluation, values ≤ 0.05 for the RMSEA and ≥0.95 for CFI and TLI were considered as indications of good fit.

Before evaluating the relationship between Part 1 (i.e., sentinel events) and Part 2 (i.e., *content* and *context* factors) of the I-Check, ICC coefficients were calculated and evaluated for all the constructs under investigation. Given a relatively small number of average homogeneous groups per organization and in case of the substantial variability of both sentinel events and content and context factors located at the organizational level, further analyses should be conducted at the level of companies rather than at the homogenous group level; specifically, zero-order correlations with the total score of sentinel events and a ‘risk balance’ indicator (i.e., the number of times in which organizations indicated in sentinel events the answer category ‘increased’ minus those indicating ‘decreased’) should be examined at the organizational level. For this purpose, such correlations was evaluated in light of Cohen’s guidelines (Cohen, 1988) for the interpretation of the magnitude of the effect size (i.e., small correlations are below | 0.30|, medium correlations range between | 0.30| and | 0.50|, while high correlations are > | 0.50| ).

As a further step for examining the validity of the I-Check, organizations were classified into different levels of risk on the basis of the total score in sentinel events, following the cut-offs proposed by INAIL (2013, p. 49), positing three different levels of work-related risk: low, medium and high. Thus, such categorisation of work-related stress was used to discriminate between content and context factors in the context of a one-way MANOVA analysis. Finally, two different one-way MANOVAs were conducted in order to investigate whether the size of organizations and the economic sectors related to their core business are discriminated by content and context factors. Given the large number of homogenous groups implied in the above analyses, a conservative alpha level of 0.001 was established to determine the statistical significance of both multivariate and univariate tests. Partial eta squared was used as a measure of effect size of the different to evaluate both multivariate and univariate effects. Consistent with Cohen (1988), partial eta squared of 0.01, 0.06, and 0.14 are considered as indicative of, respectively, small, medium, and high effect size.

Analyses conducted within the structural equation modeling framework were carried out with M*plus* 8 (Muthén and Muthén (1998–2017)), while preliminary statistics, non-linear canonical correlation analyses, correlations and group differences were examined with SPSS 25.0 (IBM Corp, 2017).

## Results

### Descriptive Statistics

Supplementary Table S2 present percentages associated with each sentinel events. Also the percentages associated with each answer category of item *content* and *context* indicators are presented in the Supplementary Table S3.

### Formative Measurement Model of Content and Context Factors

As described above, following Step 1 of the approach of Treiblmaier et al. (2011), for each dimension the item pool was split into two maximally correlated composites that were optimally weighted by means of canonical coefficients obtained by a series (one per dimension) of non-linear canonical correlation analyses. The splitting scheme and the canonical weights are reported in the Supplementary Table S4. Since the average ICC of the P components and the single variables of *Career* and *Relationships* was 0.43 (*SD* = 0.17) and the overall *Deff* was 1.96, suggesting a non-ignorable clustering effect, the multilevel confirmatory factor model discussed above can be considered an appropriate strategy to investigate the posited measurement model. Since all latent variables were defined by a single or two indicator(s), all factor loadings were fixed to unity for model identification purposes. This strategy allows to interpret the correlation among the two manifest indicators of the latent variable as fully captured by the latent variance (see Jak, 2014). Moreover, to avoid issues with model convergence (Heck and Thomas, 2015) residual variances of *Career* and *Relationships* single indicators were fixed at zero both at group and organizational levels. After a first model run, three residual variances were negative but not significantly different from zero (specifically, those associated with the second P component of *Schedule* both at the homogenous group and organizational level, and the one associated with the second P component of *Role* at the organizational level). This phenomenon is not uncommon in multilevel confirmatory factor analysis (see Heck and Thomas, 2015). Accordingly, the model was respecified by fixing to zero these parameters: this approach is consistent with the default used in other structural equation modeling softwares (e.g., EQS 6, see Bentler, 2006). Overall, the final model fit was good χ^2^_[N_within_ = 5,301,NbetweeN = 1,631, *df* = 203]_ = 633.689, *p* < 0.001, RMSEA = 0.020, CFI = 0.967, TLI = 0.950, SRMR_within_ = 0.024, SRMR_between_ = 0.061. Moreover, level specific RMSEA and CFI were, respectively, 0.021 and 0.968 for the group level and 0.031 and 0.957 for the organizational level.

**FIGURE 2:**
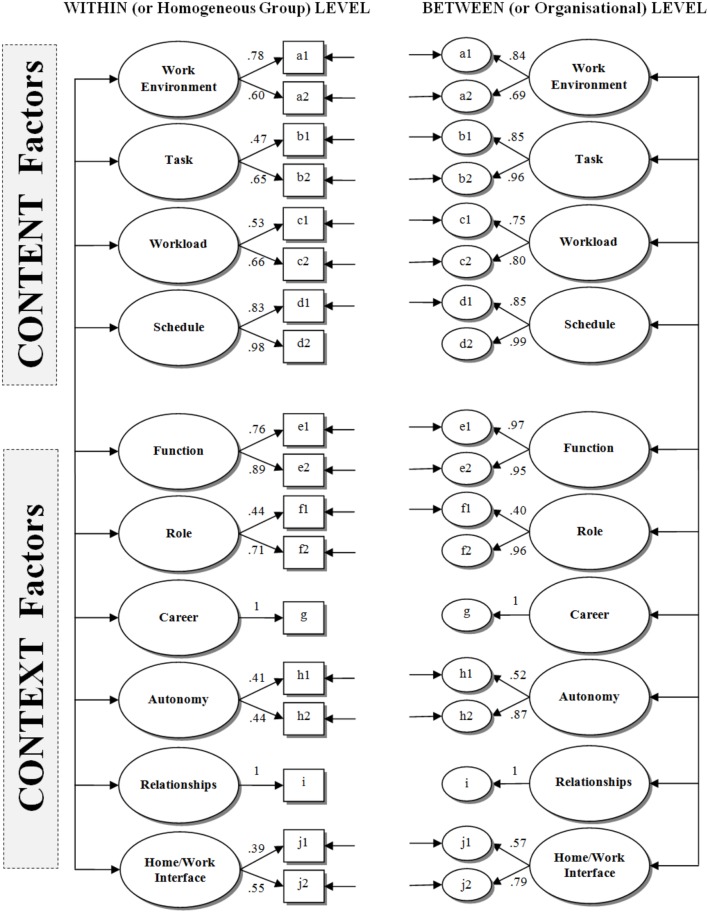
Completely standardized factor loadings from the final multilevel factor model. For sake of clarity, estimates of residual variances and latent correlations were not presented. Residual variances that are not shown in the figure were fixed to zero.

Figure 2 presents standardized factor loadings at both levels. As can be noted, factor loadings range from 0.39 to 0.98 at the group level and from 0.40 to 0.99 at the organizational level. Factor intercorrelations for both levels are presented in Supplementary Table S5. Average latent correlation among *content* factors was 0.24 at the group level (*SD* = 0.18) and 0.39 at the organizational level (*SD* = 0.22), while 0.20 (*SD* = 0.12) and 0.25 (*SD* = 0.16) were, respectively, the average latent correlations among *context* factors at the group and the organizational level. Furthermore, the average correlation between *content* and *context* factors was 0.13 (*SD* = 0.15) and 0.12 (*SD* = 0.22), respectively, at the group and organizational level.

### Relationship of Content and Context Factors With Sentinel Events

The ICC was calculated for both the composite derived from the sum of sentinel events and for *content* and *context* factors to determine if they could be aggregated at the organizational level. While the ICC for the composite of sentinel events was 0.50, results for *content* and *context* factors ranged from 0.28 (*Schedule*) to 0.76 (*Function*), suggesting that in all cases a relevant part of their total variability was located at the organizational level (Bliese, 2000). More specifically, on the one hand half of the variability of sentinel events was located at the organizational level, while on the other hand *content* scores were mainly attributable to the group level (Average ICC was 0.39), and *context* scores were mainly located at the organizational level (Average ICC was 0.56). Thus, all the scores were aggregated at the organizational level by averaging across companies (Bliese, 2000).

Table 2 presents the correlations of *content* and *context* factors with the total raw score on sentinel events and the *risk balance* (i.e., the number of times an organization declared an increased risk respect to sentinel events minus the ones in which the ‘decreased’ category was indicated). As can be noted, *content* factors, and in particular *Task*, were associated with these two indicators of work-related risk for stress, although in terms of effect size these coefficients were rather weak. The association of *context* factors with risk indicators was very low, although *Role* and *Autonomy* showed a weak but significant association with the considered indicators. Finally, *Function* and *Career* showed trivial negative correlations with sentinel events.

**Table 2:** Zero-order correlations between content and context factors with sentinel events.

	SE TOTAL RAW SCORE	SE RISK BALANCE
(1) Work environment	0.187**	0.177**
(2) Task	0.337**	0.281**
(3) Workload	0.261**	0.235**
(4) Schedule	0.258**	0.221**
(5) Function	-0.052*	-0.051*
(6) Role	0.222**	0.179**
(7) Career	-0.063*	-0.064*
(8) Autonomy	0.233**	0.202**
(9) Relationships	0.103**	0.082**
(10) Home/Work Interface	0.044	0.065**


*Content factors (upper part of the table) are separated from context factors (lower part of the table) by a dotted line. SE TOTAL RAW SCORE, Total raw sum of sentinel events scores; SE RISK BALANCE, Number of times the homogeneous groups have reported the “increased” (or *yes*) category minus those they have reported “decreased” (or *no*) in sentinel events. ^∗∗^*p* < 0.01; ^∗^*p* < 0.05.*

**FIGURE 3:**
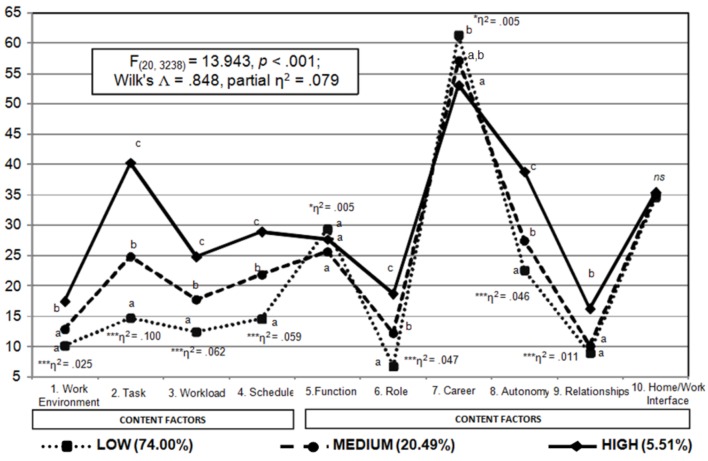
MANOVA results considering *content* and *context* factors as dependent variables and the level of risk associated with sentinel events as factor. For sake of simplicity in the interpretation, raw scores on *content* and *context* factors were rescaled on a 0–100 metric. Different superscripts denote significant differences between groups (e.g., the mean of one group is subscripted with ^a^ is significantly lower with those labeled with ^b^, etc.). Significant principal effects are the ones indicated with asterisks, where ^∗∗∗^*p* < 0.001; ^∗∗^*p* < 0.01; ^∗^*p* < 0.05, ns, not significant. η^2^ reported for principal effects refer to partial η^2^.

Applying the INAIL’s (2013) cut-offs to determine the organizational risk based on sentinel events, 1,207 organizations (74.00%) resulted at low risk, 334 organizations (20.49%) resulted at medium risk, while 90 organizations (5.51%) resulted at high risk. Figure 3 presents the different profiles of risk that were further examined via a one-way MANOVA considering *content* and *context* factors as dependent variables, and the level of risk in sentinel events derived as above as the independent variable. After ascertaining a strong multivariate effect, principal effects were scrutinized. With regards to *context* factors, Tukey’s *post hoc* test highlighted the full discrimination between risk levels (except for *Work Environment*, where organizations at low and medium risk in sentinel events did not mutually differ). In this case, principal effects were of medium size (excepting for *Work Environment*, which showed a small effect). With regards to *content* factors, similar results were found only for *Role* and *Autonomy*, although their principal effects were rather small. Moreover, organizations at high risk obtained higher scores than the ones at medium or low risk in *Relationships*, while the results for *Function* and *Career* are consistent with the pattern of correlations reported in Table 2, where low risk organizations in sentinel events obtained higher scores. However, these effects are trivial. Finally, the risk variable did not discriminate in *Home/Work Interface* scores.

Overall, risk levels detected on the basis of the scores on sentinel events revealed a consistent pattern of differences in all *content* factors, that is different levels of risk detected on the Area 1 of the I-Check reflect the same pattern of differences in all *content* dimensions with the exception of *Work Environment*, where organizations at low or medium risk in sentinel events were not found to mutually differ. In the case of *context* factors, similar results were found only for *Role* and *Autonomy* and, partially, for *Relationships*.

### Relations With Organizational Variables

Results of the one-way MANOVA considering the *size of the company* as the independent variable, indicated a significant, medium multivariate effect *F*(50,6657) = 12.407, *p* < 0.001; Wilk’s Λ = 0.666, $\eta_{p}^{2}$ = 0.078, suggesting differences between differently large companies in work *content* and *context* factors. The analysis of principal effects (Table 3) revealed a systematic tendency of smaller organizations to report lower scores in *content* factors, as well as for *Role* and *Autonomy* for *context* factors. No differences were found between organizations of different size for *Relationships*, and for *Function* and *Career* larger organizations seem to perform better than smaller ones.

**Table 3:** Principal effects of MANOVA positing the size of the company as independent variable.

	F	*df*	*df*	*p*	Partial	1–9 emp	10–50 emp	51–100 emp	101–250 emp	251–1000 emp	>1000 emp
			error		η^2^	(36.9%)	(34.4%)	(9.5%)	(7.6%)	(6.0%)	(5.6%)
(1) Work Environment	5.708	5	1468	<0.001	0.019	9.55^a^	11.06^a,b^	13.24^a,b^	13.83^b^	13.41^a,b^	14.09^b^
(2) Task	45.364	5	1468	<0.001	0.134	11.79^a^	17.31^a,b^	24.20^c^	23.13^b,c^	25^c^	43.32^d^
(3) Workload	24.082	5	1468	<0.001	0.076	10.14^a^	15.06^b^	17.38^b,c^	18.66^b,c^	20.39^c^	20.91^c^
(4) Schedule	41.371	5	1468	<0.001	0.124	10.24^a^	18^b^	23.03^b,c^	26.99^c^	24.93^c^	26.14^c^
(5) Function	16.844	5	1468	<0.001	0.054	33.96^c^	26.57^b^	23.81^a,b^	23.39^a,b^	18.59^a^	22.07^a,b^
(6) Role	12.509	5	1468	<0.001	0.041	4.53^a^	9.94^b^	12.21^b^	10.40^b^	10.17^b^	12.99^b^
(7) Career	17.564	5	1468	<0.001	0.056	65.03^b^	62.79^b^	58.22^b^	59.24^b^	40.70^a^	35.59^a^
(8) Autonomy	17.175	5	1468	<0.001	0.055	19.89^a^	26.07^b^	29.19^b,c^	26.52^b,c^	32.47^c^	32.53^c^
(9) Relationships	0.464	5	1468	0.803	0.002	8.66^a^	9.00^a^	9.28^a^	7.70^a^	10.77^a^	9.64^a^
(10) Home/Work Interface	2.511	5	1468	0.028	0.008	34.08^a,b^	36.06^b^	36.48^b^	34.05^a,b^	31.57^a,b^	26.24^a^


*Content factors are separated from context factors within the table by a dotted line. Different superscripts denote significant differences between groups (e.g., the mean of one group is subscripted with ^*a*^ is significantly lower with those labeled with ^*b*^, etc.). 1–9 emp, from 1 to 9 employees; 10–50 emp, from 10 to 50 employees; 51–100 emp, from 51 to 100 employees; 101–250 emp, from 101 to 250 employees; 251–1,000 emp, from 251 to 1,000 employees; >1,000 emp, more than 1,000 employees.*

When considering the economic *sector of the company* as an independent variable there are multivariate significant (medium) differences in *content* and *context* factors: *F*(90,10943) = 11.363, *pc* < 0.001; Wilk’s Λ = 0.546, $\eta_{p}^{2}$ = 0.065. Principal effects are reported in Table 4, suggesting a tendency to report higher scores for work *content* factors from organizations of the health and public sectors and a more complex pattern of differences for *context* factors.

**Table 4:** Principal effects of MANOVA positing economic sector as independent variable.

	F	*df*	*df*	*p*	partial	AGRI	MANU	CONST	WHOL	TRANSP	INFOR	PROF	PUBLIC	HEALTH	OTHER
			error		η^2^	(1.7%)	(22.7%)	(11.2%)	(14.0%)	(2.9%)	(4.2%)	(16.3%)	(11.4%)	(10.1)	(5.5%)
(1) Work Environment	15.418	9	1621	<0.001	0.079	20.90^e^	11.36^a,b,c^	14.78^c,d^	7.65^a,b^	12.10^b,c,d^	6.11^a^	8.11^a,b^	11.92^b,c,d^	16.82^d,e^	8.00^a,b^
(2) Task	32.524	9	1621	<0.001	0.153	9.65^a^	14.52^a,b^	11.33^a^	9.84^a^	14.31^a,b^	18.84^a,b^	20.82^b^	37.94^c^	23.08^b^	14.42^a,b^
(3) Workload	11.916	9	1621	<0.001	0.062	10.18^a,b^	15.74^b,c,d^	14.10^a,b,c,d^	8.49^a^	17.71^c,d^	9.14^a^	12.91^a,b,c^	17.28^c,d^	19.54^d^	13.29^a,b,c^
(4) Schedule	24.752	9	1621	<0.001	0.121	11.90^a^	18.23^a,b^	11.02^a^	12.73^a^	19.89^b^	12.76^a,b^	13.30^a,b^	17.21^a,b^	32.72^c^	16.61^a,b^
(5) Function	10.663	9	1621	<0.001	0.056	33.06^b,c^	29.10^b,c^	27.19^a,b,c^	35.83^c^	24.91^a,b^	27.63^a,b,c^	33.07^b,c^	24.34^a,b^	17.79^a^	25.36^a,b^
(6) Role	4.920	9	1621	<0.001	0.027	4.97^a^	8.13^a,b^	6.97^a,b^	4.72^a^	10.90^a,b^	7.34^a,b^	8.99^a,b^	12.14^b^	12.69^b^	6.65^a,b^
(7) Career	16.555	9	1621	<0.001	0.084	71.28^b^	63.12^b^	58.53^b^	69.09^b^	61.71^b^	64.43^b^	65.17^b^	33.35^a^	58.29^b^	62.23^b^
(8) Autonomy	5.785	9	1621	<0.001	0.031	22.53^a,b^	28.65^b^	24.14^a,b^	19.90^a^	28.14^a,b^	24.01^a,b^	20.51^a,b^	27.24^a,b^	24.35^a,b^	24.49^a,b^
(9) Relationships	2.046	9	1621	0.031	0.011	13.26^a^	8.78^a^	7.57^a^	9.03^a^	7.31^a^	12.66^a^	11.55^a^	11.01^a^	9.62^a^	6.33^a^
(10) Home/Work Interface	5.752	9	1621	<0.001	0.031	42.55^c^	39.87^b,c^	38.70^b,c^	35.06^a,b,c^	37.60^b,c^	23.26^a^	31.63^a,b,c^	28.75^a,b^	31.98^a,b,c^	36.01^b,c^


*Content factors are separated from context factors within the table by a dotted line. Different superscripts denote significant differences between groups (e.g., the mean of one group is subscripted with ^*a*^ is significantly lower with those labeled with ^*b*^, etc.). AGRI, Agriculture, forestry and fishing; MANU, Manufacturing; CONST, Construction; WHOL, Wholesale, retail trade, accommodation and food service activities; TRASP, Transportation and storage; INFOR, Information and communication, financial and insurance activities, real estate; PROF, Professional, scientific and technical activities; Activities of extraterritorial organizations and bodies; PUBLIC, Education, public administration and defense, compulsory social security; HEALTH, Human health and social work activities; OTHER, Other service activities.*

Overall, while *content* factors showed medium or large differences among organizational size and economic sectors (with the exception of *Work Environment*, where in the case of organizational size differences showed a small principal effect), the effect size associated with principal effects of *context* factors were generally small.

## Discussion

This study examined the psychometric characteristics of the I-Check, a checklist developed within INAIL’s methodology for the assessment and management of risks associated with work-related stress. INAIL’s web platform allowed an in-depth investigation to be conducted on the characteristics of the I-Check for a wide sample of Italian companies using this tool in a real risk assessment phase. Therefore, a first aim of this study was to investigate the psychometric properties of the I-Check, with a particular emphasis on the measurement model of the work content and of the work context factors. The developmental process of the checklist suggested to adopt a data analytical approach based on causal (or formative) indicators as the best fitting to the purpose of this study. Since the I-Check is used on clustered data (with homogenous groups of workers nested within companies), the formative measurement model was implemented posting a two-level measurement model. The results evidenced an overall satisfying fit with observed data. Moreover, when considering fit indices specific to each level of analysis, no substantial misspecifications were detected. In a nutshell, the results suggest that the tested model reached the strong configural isomorphism (Tay et al., 2014), meaning that “the factor structure of lower- and higher-level constructs are similar” (Tay et al., 2014, p. 84). Thus, the I-Check has shown adequate psychometric properties when the measurement model for *content* and *context* factors was specified considering their indicators as formative: thus, the 10 composite scores derived from the formative-indicators measurement model can be considered as valid measures of the risk factors they intend to gauge. Nevertheless, some differences in the results related to the *content* and *context* factors were found that lead to some reflections on future developments.

On the one hand, content factors showed a higher number of significant factor correlations than *contex*t factors, both at the group and organizational level. On the other hand, the average correlation among *content* and among *context* factors was small in both cases with the exception of some dimensions (i.e., *Work Environment*, *Workload* and *Schedule* with *Home/Work Interface*; *Role* with *Task*). Overall, these results suggest that *content* and *context* factors capture different aspects of risk for work-related stress, and they show a relevant degree of mutual independence. This result recommend the use of the I-Check as an instrument for deriving fine-grained organizational risk profiles rather than a broad and unique measure of “risk-of-work-related-stress” of the organization. Overall, the main aim of a risk assessment is to collect useful information on the risk level in order to put in place corrective and preventive measures, thus having organizational risk profiles may increase the capacity of the tool to capture aspects needing for improvement. The implications of this are highlighted below.

Moreover, while the variability of *content* factors was prevalently associated to the homogeneous group level, *context* factors showed a higher proportion of variability due to organizational level differences. Such results can be interpreted through the lens of an interactionist perspective, as a higher degree of shared meanings people give to the contextual aspects in an organization (Schneider and Barbera, 2014) rather than the work *content* factors which are generally conceived as more specific elements linked to the features and tasks of a specific job (Johns, 2006). Thus, it may be the case that aspects related to the context of work are more referred to at the organizational level than group level since they have common and transversal meanings for the organizations as a whole (e.g., aspects related to function and culture or career advancement criteria).

In relation to the second aim of this paper, some reflections on the relationship of *content* and *context* factors with sentinel events as objective markers of work-related stress require attention. At the organizational level, *content* factors are weakly but significantly correlated with the total score in sentinel events. On the other hand, *context* factors resulted in lower or no correlations with sentinel events, with only *Role*, *Autonomy*, and *Relationships* exhibiting some degree of association. These results were clearer when examining organizations in terms of different levels of risk derived from the total score of sentinel events by adopting cut-offs provided by INAIL (2013). While this differentiation fully discriminates *content* factors (except for *Work Environment*) where organizations at low and medium risk in sentinel events have substantially equivalent scores, similar results were found only for *Role* and *Autonomy* when investigating *context* factors. These results highlight that, at the organizational level, *content* factors showed to vary consistently across risk profiles derived from the sentinel events, while *context* factors seem to be mainly independent from them. Overall, these findings suggest a moderate degree of convergence between risk profiles identified in the Area 1 of the I-Check and *content* factors, while weaker associations were found with most of *context* factors.

Sentinel events are acknowledged as outcomes of work-related stress in the literature (Michie and Williams, 2003; Dembe et al., 2005; Eurofound and EU-OSHA, 2014) that are measured as objective markers/signals of a possible manifestation of work-related stress. Differently, work content and work context factors are acknowledged as causes of work- related stress (Bongers et al., 1993; Siegrist and Wahrendorf, 2009; Nieuwenhuijsen et al., 2010; Kivimäki et al., 2015) and are measured through verifiable indicators filled in by a steering group. Thus, we expected a correlation among such variables given their common link with work related stress risk that is the object of measure of the I-Check. However, establishing a clear “causal” relationship among sentinel events and *content* and *context* factors is beyond the scope of the present study. In this regard, we should bear in mind that the sentinel events do not refer to the same period as the I-Check section evaluating content and context factors, but rather as trends of the last 3 years.

Sentinel events were not included in I-Check as possible outcomes of the context and content factors, but as *signals* of illness likely due to stress. In particular, they are indicators of trends, aimed at assessing changes in the risk of stress in the organization. Work content and work context factors in their turn, being considered in the literature as possible causes of work-related stress, are measured in the I-Check in order to define possible interventions to be implemented in order to reduce stress. In this way, organizations using the I-Check have an assessment of work related stress risk (the sentinel events) and a roadmap for interventions (the work content and work context factors). This because they may verify the effective presence of a general illness likely linked to work stress through some objective records that are signals of that (sentinel events) and, on the other hand, they can investigate which are the aspects of work content and work context bad managed to identify interventions. In doing this it is not assumed a causal relationship among work content and work context factors and sentinel events: by the way, one of the aims of this study was to evaluate the correlation among these variables since all of them contribute in measuring the risk of work related stress, and to register that these two sets of variables “move in the same direction.”

Moreover, some aspects related to the context factors explored in the I-Check are by nature more susceptible to subjective observations and personal evaluations by the steering group who compiles the I-Check. As an example, saying that there are defined career advancement criteria is not necessarily equivalent to saying that these are applied successfully, and this may create a certain degree of ambiguity in the answers that might account for the presence of the marginal findings found for the *content* factors. We believe that this is an unavoidable part of any assessment strategy. In this regard INAIL offers a comprehensive assessment approach which integrates different tools and different assessment perspectives with the aim of managing work-related stress effectively. Of particular importance is the involvement of workers and/or their representatives in compiling the I-Check: this has been demonstrated to be essential for the effectiveness of the risk assessment, particularly for the *context* factors, since workers are the best informants as regards their working conditions and work context. In this regard, a previous study has shown that there are significant differences in results of the application of the I-Check when workers are involved in the compilation of this instrument (Di Tecco et al., 2015). Therefore, whether the validity of the tools used is fundamental for the effectiveness of the risk assessment, the process and ways the risk assessment is conducted are crucial too.

Finally some important differences also emerged regarding the organizational variables considered. Risks related to *content* factors tend to increase with the size of the companies. In other words, organizations with more employees tend to exhibit higher levels of risk associated with job content. With regards to *context* factors, similar results were found for *Autonomy* and partially for *Role*, where organizations with less than ten employees scored lower than the others. These results are in line with previous findings (Buckley, 2016) and may be also explained by the growing complexity in the organization of work linked to the increase in companies’ dimensions. An inverse pattern was shown by *Function* and *Career*, whereby smaller companies tend to score higher than bigger ones. Such a result may be due to the tendency of smaller companies to a misinterpretation of the *Function* and *Career* indicators as a way to indicate the absence of some aspects within the homogenous group (or the impossibility of evaluating them). For example, the *Function* item ‘Diffusion of an enterprise security management system’ or the *Career* item ‘Defined career advancement’ may not necessarily have the same meaning in a small company (up to 10 employees) as in a big one (over 1,000 employees); thus, in this case, indicating that they are absent may not necessarily correspond to a risk. Moreover, *Function* and *Career* are dimensions, more than the others, describing internal policies, procedures and management systems and it is likely that those items might be not completely appropriate measures in small companies (up to 10 employees) where it might be generally more difficult having written policies and procedures due to their unstructured organizational nature.

Regarding the economic sectors of the companies considered for the present study, the scores of *content* factors tend to be higher in public administration and healthcare work areas, while the differences that emerged for *context* factors are less interpretable, since economic sectors seem to weakly discriminate within their scores. The differences emerged have particularly sense in the healthcare sector where aspects related to the content of work are more critical due to the nature of the work itself: high presence of work shift and night shift, presence of other risks for health and safety (ergonomic and biological risks), lack of resources, job frequently interrupted, presence of emergencies.

### Practical Implications and Future Improvements

Valid and easy to use tools for the assessment of psychosocial risks are essential to target the problems in workplaces and put in place effective interventions, since each workplace requires distinctive solutions to manage workers’ health and safety. The findings of this study may contribute to filling in the gaps of other methodologies which provide mainly self-reporting tools while calling for collecting objective and observational indicators without offering any specific tool.

This study highlights some positive as well as critical features related to the checklist offered by INAIL’s methodology for the assessment and management of risks associated with work-related stress. Among the positive aspects is the evidence of the psychometrics qualities of this tool. In line with previous tests and studies, the findings confirm the high quality of the I-Check in a large sample of Italian companies (over 5,000). This addresses the criticisms regarding the lack of validity studies (e.g., Balducci, 2015; Corradini et al., 2016). Results of this study confirm and further expands what obtained in the previous validations conducted on smaller samples (Persechino et al., 2013; Di Tecco et al., 2015; Ronchetti et al., 2015). In light of the last 5 years of experience and the collection of wide data on companies using the INAIL methodology through the free access web platform, this study can now confirm the usefulness and validity of the I-Check as a part of the assessment of risks associated with work-related stress.

The study’s findings also represent a useful basis for improving the tool itself since some weaknesses also emerged. In particular, the non-optimal findings related to the *context* factors encourage reflection and contribute to moving toward the development of tools tailored to the specificities of different sectors, as already planned in the research agenda of INAIL. Some *context* factors (such as *Career* and *Relationships*) may in fact be more susceptible to these specificities than the *content* factors, and, may require further integration in terms of items comprising the checklist. Since some sectors emerged as more at risk (namely Health Care and Public Administration), INAIL is now working on the adaptation of the I-Check in a “tailor-made” perspective using a participative research approach. The results of this work will be offered to the companies on INAIL’s web platform in the next months.

Further practical implications are related to the scoring system of the I-Check. First, the findings show a relevant degree of mutual independence between *content* and *context* factors, suggesting the adoption of a risk profile approach for the scoring instead of a total score for the three areas of the I-Check. Although the total score is quite useful for identifying a general risk level, the possibility of deriving “risk profiles” would be even more recommendable in order to increase the capacity of the tool in capturing specific aspects of work-related stress and in supporting the identification of the best fitting interventions do deal with aspects. Moreover, the amount of data collected will also allow the scoring system to be brought up to date for more than 5,000 groups using updated percentiles. The revised scoring system is included in the new 2017 edition of the user guide of the INAIL’s methodology (INAIL, 2017).

### Limitations

Although we conducted this study at our best, we acknowledge it has some limitations.

The study has been conducted in a specific European country, Italy, and this apparently represents a limitation for the generalization of the results to other countries. However, the development of the I-Check aims at filling a gap in the work-related stress assessment, above and beyond the use of this specific instrument in a specific country. As noted in the introduction, the absence of a standardized tool for the measurement of objective and organizational data related to work-related stress is far from being specific to the Italian case. As a matter of fact, internationally used methodologies and frameworks, such as the British Heath and Safety Executive (HSE), while calling from this type of instruments, do not provide any tool that can be used in practice. It is obvious, by the way, that claims for the generalisability of our results must come from the use of the I-Check in cultural contexts other than the Italian.

Organizations that used the preliminary assessment of INAIL’s methodology (INAIL, 2013) are not fully statistically representative of the Italian organizational population in terms of economic sectors and size (ISTAT, 2016), since the final data reflected evaluations expressed by the steering groups of those organizations that voluntarily chose to adopt the INAIL methodology. Thus, notwithstanding the wider use of INAIL’s methodology among Italian companies, which has emerged as the most used methodology in Italy, the present findings cannot be fully generalized to Italian organizations overall.

With regard to the composition of our sample, the high presence of small-size companies (about 77% are micro and small companies) might constitute a further limitation; however, this aspect is closely connected to Italian organizations composition. In Italy, about the 95% of companies are micro and small businesses (see ISTAT, 2016), and this fact may reflect a potential barrier for the cross-cultural generalization of our findings to other enterprise systems. Therefore, our sample reflects this composition in relation to the organizational size, although in a not fully representative way. With respect to this limitation, we want also emphasize that our unit of analysis is the homogeneous group (and not the organization), that are identified with the same criteria both in small and large organizations, so the size and the number of homogeneous groups in terms of employees is proportional to the size of the organization.

This study is cross-sectional, so that evaluations expressed by the steering groups focused on a specific organization phase referring to a given time point. This is due to the fact that INAIL’s methodology has been available from 2011, thus most companies are likely now working on completing the second round of assessment. Further studies should incorporate multiple time points of assessment to evaluate the stability of work-related risk. This approach may be useful especially after corrective interventions, since it can be used to evaluate their effectiveness in adopting specific research designs (Van der Klink et al., 2001; Nielsen and Miraglia, 2017).

The present study focused on the validation of the preliminary assessment phase of INAIL’s overall methodology. As noted above, since the Area 1 of the I-Check (i.e., sentinel events) refers to the modification of some organizational markers of work-related stress within the time frame of the previous 3 years, it is impossible to establish its “causal” relationship with *content* and *context* factors. With the aim to fill this gap, the new INAIL’s platform (INAIL, 2017) allows to record explicitly the information to calculate each sentinel event (e.g., nominal percentage of sick leave absences for the previous year and for the past 3-year period) and to track the preliminary assessment of the INAIL’s methodology along multiple time points. Such improvements will allow for a deeper understanding of the I-Check functioning and the association among its areas in the near future.

Finally, although *content* and *context* factors were also investigated in terms of their relationship with sentinel events, no other external criteria were included. Further studies should incorporate potential organizational outcomes of the work-related stress process, such as burnout, job satisfaction, health symptoms and well-being, possibly collected from sources different from the steering group.

## Author Contributions

CB contributed on all sections of the paper. In particular he took care of the theoretical framework, to the literature review, to the methods, and to the definition of the data analytical strategy. VG and RF contributed to the methods and data analysis. CDT, MR, MG, BP, and SI contributed on the literature review and to the institutional framework review. All the authors added their contribution to the writing of the sections of Discussions and Conclusion. VG and CDT equally contributed to this work.

## Conflict of Interest Statement

The authors declare that the research was conducted in the absence of any commercial or financial relationships that could be construed as a potential conflict of interest.

## References

[B1] Allisey A. Rodwell J. Noblet A. (2016). An application of an extended effort-reward imbalance model to police absenteeism behaviour. *Pers. Rev.* 45 663–680. 10.1108/PR-06-2014-0125

[B2] Balducci C. (2015). *Gestire lo Stress Nelle Organizzazioni. Psicologia in Pratica – Lavoro e Organizzazione.* Bologna: Il Mulino.

[B3] Bentler P. M. (1990). Comparative fit indexes in structural models. *Psychol. Bull.* 107 238–246. 10.1037/0033-2909.107.2.238 2320703

[B4] Bentler P. M. (2006). *EQS 6 Structural Equations Program Manual.* Encino, CA: Multivariate Software, Inc.

[B5] Bliese P. D. (2000). “Within-group agreement, non-independence, and reliability: Implications for data aggregation and analysis,” in *Multilevel Theory, Research, and Methods in Organizations: Foundations, Extensions, and New Directions*, eds Klein K. J. Kozlowski S. J. Klein K. J. Kozlowski S. J. (San Francisco, CA: Jossey-Bass), 349–381.

[B6] Bollen K. A. (1989). *Structural Equations with Latent Variables.* Oxford: John Wiley & Sons 10.1002/9781118619179

[B7] Bollen K. A. Davis W. R. (2009). Causal indicator models: identification, estimation, and testing. *Struct. Equat. Model.* 16 498–522. 10.1080/10705510903008253

[B8] Bollen K. A. Diamantopoulos A. (2017). In defense of causal-formative indicators: a minority report. *Psychol. Methods* 22 581–596. 10.1037/met0000056 26390170 PMC6670294

[B9] Bongers P. M. de Winter C. R. Kompier M. A. Hildebrandt V. H. (1993). Psychosocial factors at work and musculoskeletal disease. *Scand. J. Work Environ. Health* 19 297–312. 10.5271/sjweh.1470 8296178

[B10] Buckley P. (2016). *Work Related Stress, Anxiety and Depression Statistics in Great Britain 2016.* Bootle: Health and Safety Executive.

[B11] Cadogan J. W. Lee N. (2013). Improper use of endogenous formative variables. *J. Bus. Res.* 66 233–241. 10.1016/j.jbusres.2012.08.006

[B12] Cohen J. (1988). *Statistical Power Analysis for the Behavioral Sciences*, 2nd Edn Hillsdale, NJ: Erlbaum.

[B13] Corradini I. Marano A. Nardelli E. (2016). Work-related stress risk assessment: a methodological analysis based on psychometric principles of an objective tool. *SAGE Open* 6 1–9. 10.1177/2158244016666888

[B14] Cousins R. Mackay C. J. Clarke S. D. Kelly C. Kelly P. J. McCaig R. H. (2004). Management Standards’ and work-related stress in the UK: practical development. *Work Stress* 18 113–136. 10.1080/02678370410001734322

[B15] Cox T. (1990). “The recognition and measurement of stress: conceptual and methodological issues,” in *Evaluation of Human Work*, eds Corlett E. N. Wilson J. (London: Taylor &Francis).

[B16] Cox T. Cox S. (1993). *Psychosocial and Organizational Hazards: Monitoring and Control. Occasional Series in Occupational Health 5.* Copenhagen: World Health Organization.

[B17] Cox T. Griffiths A. (1995). “The nature and measurement of work stress: theory and practice,” in *The Evaluation of Human Work: A Practical Ergonomics Methodology*, eds Wilson J. R. Corlett N. (London: Taylor & Francis).

[B18] Dembe A. E. Erickson J. B. Delbos R. G. Banks S. M. (2005). The impact of overtime and long work hours on occupational injuries and illnesses: new evidence from the United States. *Occup. Environ. Med.* 62 588–597. 10.1136/oem.2004.016667 16109814 PMC1741083

[B19] Di Tecco C. Ronchetti M. Ghelli M. Russo S. Persechino B. Iavicoli S. (2015). Do Italian companies manage work-related stress effectively? a process evaluation in implementing the INAIL methodology. *BioMed Res. Int.* 2015:197156. 10.1155/2015/197156 26504788 PMC4609328

[B20] Diamantopoulos A. Siguaw J. A. (2006). Formative versus reflective indicators in organizational measure development: a comparison and empirical illustration. *Br. J. Manag.* 17 263–282. 10.1111/j.1467-551.2006.00500

[B21] Edwards A. Webster S. Van Laar D. Easton S. (2008). Psychometric analysis of the UK health and safety executive’s management standards work-related stress Indicator Tool. *Paper Presented at the British Academy of Management Annual Conference*, Harrogate. 10.1080/02678370802166599

[B22] EU-OSHA (2000). *Research on Work-related Stress.* Luxembourg: Publications Office of the European Union.

[B23] EU-OSHA (2015). *Second European Survey of Enterprises on New and Emerging Risks (ESENER-2).* Bilbao: European Agency for Safety and Health at Work 10.2802/113548

[B24] Eurofound EU-OSHA (2014). *Psychosocial Risks in Europe: Prevalence and Strategies for Prevention.* Luxembourg: Publications Office of the European Union 10.2806/70971

[B25] European Commission (2011). Report on the Implementation of the European social partners’ Framework Agreement on Work-related Stress, SEC (2011) 241 final. Brussels: European Commission.

[B26] European Trade Union Congress (2004). *European Framework Agreement on Work Related Stress.* Brussels: ETUC.

[B27] European Trade Union Congress (2007). *Framework Agreement on Harassment and Violence at Work.* Brussels: ETUC.

[B28] Hacker W. (1991). Objective work environment: analysis and evaluation of objective work characteristics. *Paper Presented to a Healthier Work Environment: Basic Concepts & Methods of Measurement*, Stockholm.

[B29] Hacker W. Iwanova A. Richter P. (1983). *Tatigkeits-bewertungssystem (TBSL).* Gottingen: Hogrefe.

[B30] Heck R. H. Thomas S. L. (2015). *An Introduction to Multilevel Modeling Techniques: MLM and SEM Approaches using Mplus*, 3rd Edn New York, NY: Routledge 10.4324/9781315746494

[B31] Hox J. J. (2010). *Multilevel Analysis: Techniques and Applications*, 2nd Edn New York, NY: Routledge 10.4324/9780203852279

[B32] Hu L. Bentler P. M. (1999). Cutoff criteria for fit indexes in covariance structure analysis: conventional criteria versus new alternatives. *Struct. Equat. Model.* 6 1–55. 10.1080/10705519909540118

[B33] Iavicoli S. Natali E. Deitinger P. Rondinone M. B. Ertel M. Jain A. (2011). Occupational health and safety policy and psychosocial risks in Europe: the role of stakeholders’ perceptions. *Health Policy* 101 87–94. 10.1016/j.healthpol.2010.08.005 20832135

[B34] Iavicoli S. Natali E. Ghelli M. Cafiero V. Mirabile M. Persechino B. (2009). Esperienze europee in tema di rischi psicosociali. *G Ital Med Lav Erg* 31 265–269. 19943440

[B35] IBM Corp (2017). *IBM SPSS Statistics for Windows, Version 25.0.* Armonk, NY: IBM Corp.

[B36] INAIL (2013). *Managing and Assessing the Risk for Work-Related Stress. Guide for Companies, in Compliance with Leg. Decree 81/2008 and Subsequent Integrations and Modifications Research Area Edition.* Milan: INAIL.

[B37] INAIL (2017). *Metodologia per la Valutazione e Gestione del rischio Stress lavoro-Correlato: Manuale ad uso delle aziende in attuazione del d.lgs.* 81/2008 e s.m.i Research Area Edition Milan: INAIL.

[B38] ISTAT (2016). *Rapporto sulla competitività dei settori produttivi. Letture statistiche – Temi.* Rome: ISTAT.

[B39] Jak S. (2014). Testing strong factorial invariance using three-level structural equation modeling. *Front. Psychol.* 5:745. 10.3389/fpsyg.2014.00745 25120499 PMC4110441

[B40] Jarvis C. B. MacKenzie S. B. Podsakoff P. M. (2003). A critical review of construct indicators and measurement model misspecification in marketing and consumer research. *J. Consum. Res.* 30 199–218. 10.1086/376806

[B41] Johns G. (2006). The essential impact of context on organizational behavior. *Acad. Manag. Rev.* 31 386–408. 10.2307/20159208

[B42] Kivimäki M. Jokela M. Nyberg S. T. Singh-Manoux A. Fransson E. I. Alfredsson L. (2015). Long working hours and risk of coronary heart disease and stroke: a systematic review and meta-analysis of published and unpublished data for 603 838 individuals. *Lancet* 386 1739–1746. 10.1016/S0140-6736(15)60295-1 26298822

[B43] Lee N. Cadogan J. W. (2013). Problems with formative and higher-order reflective variables. *J. Bus. Res.* 66 242–247. 10.1016/j.jbusres.2012.08.004

[B44] Leka S. Cox T. (2008). *The European Framework for Psychosocial Risk Management.* Nottingham: PRIMA-EF. 10.1539/joh.o10010 21325735

[B45] Leka S. Jain A. Iavicoli S. Di Tecco C. (2015). An evaluation of the policy context on psychosocial risks and mental health in the workplace in the European Union: achievements, challenges, and the future. *BioMed Res. Int.* 2015:213089. 10.1155/2015/213089 26557655 PMC4628767

[B46] Leka S. Jain A. Zwetsloot G. Cox T. (2010). Policy-level interventions and work-related psychosocial risk management in the European Union. *Work Stress* 24 298–307. 10.1080/02678373.2010.519918

[B47] Mackay C. J. Cousins R. Kelly P. J. Lee S. McCaig R. H. (2004). Management Standards’ and work-related stress in the UK: policy background and science. *Work Stress* 18 91–112. 10.1080/02678370410001727474

[B48] Michie S. Williams S. (2003). Reducing work related psychological ill health and sickness absence: a systematic literature review. *Occup. Environ. Med.* 60 3–9. 10.1136/oem.60.1.3 12499449 PMC1740370

[B49] Muthén B. O. Satorra A. (1995). Complex sample data in structural equation modeling. *Sociol. Methodol.* 25 267–316. 10.2307/271070

[B50] Muthén L. K. Muthén B. O. (1998–2017). *Mplus User’s Guide* 8th Edn Los Angeles, CA: Muthén & Muthén.

[B51] Nielsen K. Miraglia M. (2017). What works for whom in which circumstances? On the need to move beyond the “what works?’ question in organizational intervention research. *Hum. Relat.* 70 40–62. 10.1177/0018726716670226 25265163

[B52] Nielsen K. Randall R. (2013). Opening the black box: presenting a model for evaluating organizational-level interventions. *Eur. J. Work Organ. Psychol.* 22 601–617. 10.1080/1359432X.2012.690556

[B53] Nielsen K. Randall R. Holten A. L. González E. R. (2010). Conducting organizational-level occupational health interventions: What works? *Work Stress* 24 234–259. 10.1080/02678373.2010.515393

[B54] Nieuwenhuijsen K. Bruinvels D. Frings-Dresen M. (2010). Psychosocial work environment and stress-related disorders, a systematic review. *Occup. Med.* 60 277–286. 10.1093/occmed/kqq081 20511268

[B55] Persechino B. Valenti A. Ronchetti M. Rondinone B. M. Di Tecco C. Vitali S. (2013). Work related stress risk assessment in Italy: a methodological proposal adapted to regulatory guidelines. *Safety Health Work* 4 95–99. 10.1016/j.shaw.2013.05.002 23961332 PMC3732139

[B56] Regulies R. (2012). Studying the effect of the psychosocial work environment on risk of ill-health: towards a more comprehensive assessment of working conditions. *Scand. J. Work Environ. Health* 38 187–192. 10.5271/sjweh.3296 22476352

[B57] Ronchetti M. Di Tecco C. Russo S. Castaldi T. Vitali S. Autieri S. (2015). An integrated approach for the assessment of work-related stress risk: comparison between findings from the tools of an Italian methodology. *Safety Sci.* 80 310–316. 10.1016/j.ssci.2015.08.005

[B58] Rondinone B. M. Persechino B. Castaldi T. Valenti A. Ferrante P. Ronchetti M. (2012). Work-related stress risk assessment in Italy: the validation study of health safety and executive indicator tool. *G Ital Med Lav Erg* 34 392–399. 23477105

[B59] Ryu E. (2014). Model fit evaluation in multilevel structural equation models. *Front. Psychol.* 5:81. 10.3389/fpsyg.2014.00081 24550882 PMC3913991

[B60] Ryu E. West S. G. (2009). Level-specific evaluation of model fit in multilevel structural equation modeling. *Struct. Equat. Model.* 16 583–601. 10.1080/10705510903203466

[B61] Salminen S. (2004). Have young workers more injuries than older ones? An international literature review. *J. Safety Res.* 35 513–521. 10.1016/j.jsr.2004.08.005 15530925

[B62] Schneider B. Barbera K. M. (2014). *The Oxford Handbook of Organizational Climate and Culture.* New York, NY: Oxford University Press 10.1093/oxfordhb/9780199860715.001.0001

[B63] Siegrist J. Wahrendorf M. (2009). Quality of work, health, and retirement. *Lancet* 374 1872–1873. 10.1016/S0140-6736(09)61666-4 19897237

[B64] Steiger J. H. (1990). Structural model evaluation and modification: an interval estimation approach. *Multivariate Behav. Res.* 25 173–180. 10.1207/s15327906mbr2502_4 26794479

[B65] Tay L. Woo S. E. Vermunt J. K. (2014). A conceptual and methodological framework for psychometric isomorphism: validation of multilevel construct measures. *Organ. Res. Methods* 17 77–106. 10.1177/1094428113517008

[B66] Treiblmaier H. Bentler P. M. Mair P. (2011). Formative constructs implemented via common factors. *Struct. Equat. Model.* 18 1–17. 10.1080/10705511.2011.532693

[B67] Van der Klink J. J. Blonk R. W. Schene A. H. Van Dijk F. J. (2001). The benefits of interventions for work-related stress. *Am. J. Public Health* 91 270–276. 10.2105/AJPH.91.2.270 11211637 PMC1446543

