# Supplementary material for: Assessing Objective and Verifiable Indicators Associated With Work-Related Stress: Validation of a Structured Checklist for the Assessment and Management of Work-Related Stress
Source: Front Psychol. 2018 Dec 4;9:2424. doi: 10.3389/fpsyg.2018.02424 (PMC6288307; doi:10.3389/fpsyg.2018.02424)
Supplement: Supplementary file 1 [file Data_Sheet_1.docx]

Supplementary Material

**Assessing objective and verifiable indicators associated with work-related stress: validation of a Structured Checklist for the Assessment and Management of Work-Related Stress**

**Claudio Barbaranelli^a^, Valerio Ghezzi^a*^, Cristina Di Tecco^b*^,**

**Matteo Ronchetti^b^, Roberta Fida^c^, Monica Ghelli^b^, Benedetta Persechino^b^, Sergio Iavicoli^b^**

*Valerio Ghezzi and Cristina Di Tecco equally contributed to this work

^a^Department of Psychology – University of Rome “Sapienza”, Rome, Italy

^b^Department of Occupational and Environmental Medicine, Epidemiology and Hygiene–INAIL, Italian National Workers Compensation Authority, Rome, Italy

^c^Norwich Business School - University of East Anglia, Norwich, United Kingdom

***Appendix A*. Formative versus Reflective Measurement Models**

Recently, Bollen and Bauldry (2011) provided an excellent update to the literature concerning the distinctiveness of formative and reflective indicators in psychological and social sciences. Both types of indicators are developed in order to correspond to the theoretical definition of the concept represented by the latent variable of which they are indicators of. However, they can be distinguished by the direction of the influence that links the indicator to the latent variable(s).While formative (or *causal*) indicators influence the latent variable directly, reflective (or *effect*) indicators are influenced directly by the latent variable. Formative indicators are linked to the latent variable by means of *structural coefficients* (i.e. the *betas* in a regression model or in a structural equation model): thus, the latent variable they measure *is caused* by the indicators themselves. Reflective indicators are linked to latent variables by means of *factor loadings*, following the assumption that the latent variables *cause* the indicators, and explains what they share. Classical test theory, item response theory, exploratory and confirmatory factor analysis, and reliability estimation assume that indicators are effect indicators. While a set of reflective indicators of a latent variable should all be correlated with one another, formative indicators do not require such an association (Bollen & Bauldry, 2011; Bollen & Diamantopoulos, 2017; Treiblmaier, Bentler, & Mair, 2011). This is a feature shared by many checklists, questionnaires and measurement instruments used in health research (e.g. Costa, 2015), organisational research (Costa & Anderson, 2011), management (Barrales-Molina*,* Bustinza, & Gutiérrez-Gutiérrez, 2013) and developmental psychology (Willoughby, Kuhn, Blair, Samek, & List, 2017).

Given these premises, a strategy based on formative indicators was considered the more compatible and suitable to model WCN and WCO factors, because indicators of these factors are not conceptually interchangeable (MacKenzie, Podsakoff, & Jarvis, 2005) and they ‘jointly determine the conceptual and empirical meaning of the construct[s]’ (Jarvis, MacKenzie, & Podsakoff, 2003).

Among the various issues raised regarding the implementation of measurement models based on formative indicators, two are particularly relevant here. The first refers to the absence of measurement error (i.e. the influence of various random factors on the precision of measurement process) in models based on causal indicators. The second is related to the possibility of estimating a model with causal indicators identifying all the model parameters: in particular, it has been noticed that when no paths are emitted from the latent variable, the model is virtually never identified (Bollen & Lennox, 1991). This renders it impossible to test formative measurement models *per se*, unless they are embedded within a more complex nomological network where these latent variables exert their influence on other latent constructs or observed variables. Moreover, as noted by Howell, Breivik and Wilcox (2007) and Kim Shin and Grover (2010), when the latent variables measured by formative indicators are specified as aiming at dependent variables, latent or observed, their meaning can be altered according to the dependent variable specified as an effect of the latent variable, that is ‘changing dependent constructs changes the formative construct’ (Howell et al., 2007).

***Appendix B*. The Treiblmaier et al. (2011) procedure**

The procedure two-step procedure devised by Treiblmaier et al. (2011) allows for the implementation of formative measurement models via common factors. In *Step 1* is devoted to the identification of maximally correlated composites for each latent variable. Treiblmaier et al. (2011), in this regard, make a clear distinction between the F formative latent variable, whose indicators are the observed variables V_ij_, and the P *composite variable* obtained by the weighted sum of V_ij_ so that F = Σ b_ij_V_ij_ + D = P+D, where D is a random disturbance (see Panel 1 of Figure 1) uncorrelated with V_ij_ and hence with P. As demonstrated by the authors, given the P composite variable of weighted sum of V_ij_ formative indicators of a F latent variable, it is possible to decompose P into two or more parts so that P = P_1_+P_2_…_._+P_n_. Figure 1 shows the diagrammatical representation of this procedure, taking the canonical formative latent variable *Role* as an example. In Figure 1, Panel 1 represents the conceptual model for the formative latent variable F while the grey shaded areas of Panel 2 represent Step 1 of the aforementioned approach of Treiblmaier et al. (2011). As can be noted, the observed variables V_ij_ represent the constituents of the two P_i_ components in which the non-random part of the latent variable F (in Figure 1, represented by *Role*) has been partitioned, and thus they are the *formative indicators* of F, while b_ij_ represent the beta weights connecting formative indicators to components P_i_ and hence to the latent variable F; it is easy, in our example, to show that F = P + D = P_1_+P_2_+D = (b_11_V_11_+b_21_V_21_) + (b_12_V_12_+b_22_V_22_) + D.

In *Step 2,* the identification of the measurement model is at issue. As noted above, a *pure* formative measurement model cannot be identified *per se*. In order to achieve identification, the approach of Treiblmaier et al. (2011) considers the composites P_1_ and P_2_ as if they were *reflective* *indicators* of an F ‘common factor’ latent variable. It is assumed that E residuals are not correlated. The F latent variable captures the variance shared by P_i_ components, thus representing a latent variable *approximation* of P (i.e. of the *determinate* or non-random part of F, as in Treiblmaier et al., 2011), so that P_1_=β_1_F+E_1_ and P_2_=β_2_F+E_2_ (see panel 2 of Figure 1). F represents a better approximation to F as far as the two weighted components identified in step 1, i.e. P_1_ and P_2_ in our figure, are highly correlated, and this implies that the two factor loadings β_1_ and β_2_ are as large as possible. Finally, the new ‘common factor’ F (which is a *reflective approximation* of the formative latent variable F, see Figure 1) can be incorporated into a measurement model with other latent variables or embedded in a more complex structural equation model. The non-grey areas in Figure 1-Panel 2 represent Step 2. As noted by the authors "Step 1 […] is simply another data preparation step, similar to those routinely used for pre-processing and data reduction, whereas Step 2 is a standard structural modelling analysis” (p. 13). Certainly, the method devised by Treiblmaier et al. (2011) represents a brilliant ”compromise in the formative–reflective controversy” (p. 13).

***Table S1*.**

**Sectors of Economic Activities and Size of the Companies Represented Within the Sample at the Organizational Level.**

| **Sectors of Economic Activities** | **Frequency** | **%** |
| --- | --- | --- |
| Agriculture, forestry and fishing | 28 | 1.72 |
| Manufacturing | 371 | 22.75 |
| Construction | 182 | 11.16 |
| Wholesale, retail trade, accommodation and food service activities | 228 | 13.98 |
| Transportation and storage | 47 | 2.88 |
| Information and communication, financial and insurance activities, real estate | 69 | 4.23 |
| Professional, scientific and technical activities; Activities of extraterritorial organizations and bodies | 266 | 16.31 |
| Education, public administration and defense, compulsory social security | 186 | 11.40 |
| Human health and social work activities | 164 | 10.06 |
| Other service activities | 90 | 5.52 |
| **Size of the Companies** | **Frequency** | **%** |
| From 1 to 9 employees | 544 | 36.91 |
| From 10 to 50 employees | 507 | 34.40 |
| From 51 to 100 employees | 140 | 9.50 |
| From 101 to 250 employees | 112 | 7.60 |
| From 251 to 1000 employees | 88 | 5.97 |
| 1001 or more employees | 83 | 5.63 |

*Note*. Sectors of economic activities are based on the Statistical Classification of Economic Activities in the European Community, rev. 2 (2008). A “collapsed” version of economic sectors previously adopted in other studies conducted by INAIL (Inail, 2014). Percentages for the sectors of economic activities are based on all available cases, while valid percentages (referring to a subsample of 1474 companies) were considered for the size of the company.

***Table S2.***

**Percentages of Answer Categories Endorsed for Sentinel Events at the Organizational Level.**

| **Label** | **Sentinel Events** | **DECREASED %** | **UNVARIED %** | **INCREASED %** |
| --- | --- | --- | --- | --- |
| ES1 | Work-related Injuries | 54.32 | 33.17 | 12.51 |
| ES2 | Sickness Absences | 34.83 | 42.67 | 22.50 |
| ES3 | Absences from work | 36.60 | 45.19 | 18.21 |
| ES4 | Left-over vacation days | 38.20 | 44.39 | 17.41 |
| ES5 | Job Rotation | 48.25 | 46.35 | 5.40 |
| ES6 | Turnover | 40.65 | 39.24 | 20.11 |
| ES7 | Legal actions/disciplinary sanctions | 56.10 | 36.11 | 7.79 |
| ES8 | Requests for extraordinary visits | 57.76 | 37.83 | 4.41 |
|  |  |  | **YES** | **NO** |
| ES9 | Formal records of employees | - | 5.52 | 94.48 |
| ES10 | Notifications | - | 5.03 | 94.97 |

# *Table S3*.

# Percentages of Answer Categories Endorsed for the Indicators of Content and Context Factors (Homogeneous Group Level).

| **CONTENT FACTORS** | | | |
| --- | --- | --- | --- |
| **1. Work Environment and Work Equipment (*Work Environment*)** | | **NO** | **YES** |
| 1. | Noise Exposure exceeding the second level of action | 92.40 | 7.60 |
| 2. | Inadequate acoustic comfort (non-industrial environments) | 92.15 | 7.85 |
| 3. | Cancer/chemical risk not irrelevant | 88.98 | 11.02 |
| 4. | Suitable microclimate | 15.94 | 84.06 |
| 5. | Workplace adequate lighting with particular regard to eye strain activities (i.e. CVS. visually demanding jobs) | 5.98 | 94.02 |
| 6. | Risks associated with manual handling of loads | 64.25 | 35.75 |
| 7. | Available. adequate and comfortable PPEs | 3.53 | 96.47 |
| 8. | Risk of physical assault at work/solitary work | 82.36 | 17.64 |
| 9. | Immediate. clear and risk-related safety signs | 5.68 | 94.32 |
| 10. | Exposure to exceeded levels of vibrations | 95.94 | 4.06 |
| 11. | Adequate maintenance of equipment and machinery | 6.06 | 93.94 |
| 12. | Exposure to ionizing radiation | 96.49 | 3.51 |
| 13. | Exposure to biological hazards | 77.61 | 22.39 |
| **2. Task planning (*Task*)** | | **NO** | **YES** |
| 14. | Frequent interruptions at work | 65.69 | 34.31 |
| 15. | Adequacy of equipment resources to accomplish the task | 10.81 | 89.19 |
| 16. | Particularly monotonous works | 92.30 | 7.70 |
| 17. | The work requires difference tasks at once to be performed | 59.10 | 40.90 |
| 18. | Clear definition of tasks | 7.49 | 92.51 |
| 19. | Adequate human resources to perform the tasks | 18.45 | 81.55 |
| **3. Workload – Pattern of Work (*Workload*)** | | **NO** | **YES** |
| 20. | Employees execute their tasks autonomously | 6.04 | 93.96 |
| 21. | There are unpredictable variations in the amount of job | 64.25 | 35.75 |
| 22. | Long periods of inactivity during work shift | 96.98 | 3.02 |
| 23. | Job characterized by high repeatability | 86.40 | 13.60 |
| 24. | Fixed work rate for the execution of the task | 78.17 | 21.83 |
| 25. | Employees cannot vary the rhythm of machinery | 94.00 | 6.00 |
| 26. | Workers must make quick decisions | 71.16 | 28.84 |
| 27. | The job involves the use of hazardous machinery and equipment | 87.95 | 12.05 |
| 28. | Employees assume great responsibilities for others and production facilities | 70.63 | 29.37 |
| **4. Working Hours (*Schedule*)** | | **NO** | **YES** |
| 29. | Working time usually exceeds 8 hours per day | 85.70 | 14.30 |
| 30. | Played overtime | 80.38 | 19.62 |
| 31. | Tight working schedule | 65.67 | 34.33 |
| 32. | Work schedules change frequently | 91.38 | 8.62 |
| 33. | Work breaks are clearly defined | 77.49 | 22.51 |
| 34. | Shift work | 73.85 | 26.15 |
| 35. | Night shift work | 88.19 | 11.81 |
| 36. | Fixed or rotating night shift | 87.21 | 12.79 |
| **CONTEXT FACTORS** | | | |
| **5. Function and Organizational Culture (*Function*)** | | **NO** | **YES** |
| 37. | Diffusion of the organizational chart | 13.56 | 86.44 |
| 38. | Business procedures are used | 14.22 | 85.78 |
| 39. | Company procedures are illustrated to employees | 16.26 | 83.74 |
| 40. | Company goals and objectives are shared with workers | 23.30 | 76.70 |
| 41. | Diffusion of an enterprise security management system | 40.26 | 59.74 |
| 42. | Business communications system (bulletin boards. Internet. paycheck stuffers. flyers...) | 5.79 | 94.21 |
| 43. | Meetings between management and employees | 17.26 | 82.74 |
| 44. | Training plan for the professional growth of employees | 36.24 | 63.76 |
| 45. | Company communications to all staff | 19.71 | 80.29 |
| 46. | Codes of ethics and conduct (disciplinary codes are not included) | 40.82 | 59.18 |
| 47. | Counseling for work-related hardship (stress. bullying...) | 49.82 | 50.18 |
| **6. Role Within the Organization (*Role*)** | | **NO** | **YES** |
| 48. | Employees know the company’s hierarchy structure | 2.15 | 97.85 |
| 49. | Roles are clearly defined | 4.17 | 95.83 |
| 50. | Employees have multiple overlapping roles (shift supervisor. line manager. quality manager) | 80.00 | 20.00 |
| 51. | Top/line managers provide conflicting information concerning the job | 90.55 | 9.45 |
| **7. Career Path (*Career*)** | | **NO** | **YES** |
| 52. | Defined career advancement | 59.37 | 40.63 |
| 53. | Reward systems for the proper management of employees by managers/leaders | 51.86 | 48.14 |
| 54. | Reward systems for the achievement of safety objectives | 48.31 | 51.69 |
| **8. Decisional Making - Work Control (*Autonomy*)** | | **NO** | **YES** |
| 55. | Work depends on the activities previously carried out by others | 59.67 | 40.33 |
| 56. | Employees are sufficiently autonomous to decide how to do their job | 4.43 | 95.57 |
| 57. | Employees have access to information on business decisions relating to the Working Group | 18.69 | 81.31 |
| 58. | Employees are allowed to participate in the decision-making process | 59.97 | 40.03 |
| 59. | Strict job monitoring protocols | 85.34 | 14.66 |
| **9. Interpersonal Relationships at Work (*Relationships*)** | | **NO** | **YES** |
| 60. | Employees can communicate with top managers | 2.23 | 97.77 |
| 61. | Misconduct of top managers and colleagues are properly managed | 17.96 | 82.04 |
| 62. | Reporting of conflicts and arguments frequently | 93.44 | 6.56 |
| **10. Work-Home Interface - Work-Life Reconciliation (*Home/Work Interface*)** | | **NO** | **YES** |
| 63. | Meal break in an adequate place (company canteen) | 29.22 | 70.78 |
| 64. | Offered flexible work arrangements | 37.24 | 62.76 |
| 65. | Opportunity to get to work by public transportation/company shuttle bus service | 37.63 | 62.37 |
| 66. | Opportunity to perform vertical and horizontal part-time work | 33.16 | 66.84 |

Note. INAIL’s scoring system automatically provides the same direction of scoring, so that higher scores indicate higher work-related stress risk (i.e. ‘no risk’ in one item corresponds to 0;‘at risk’ corresponds to 1).

***Table S4*.**

**Canonical Coefficients Used in Step 1 of Treiblmaier et al. (2011) Procedure.**

| Formative Construct | Label of the P component | Items belonging to the  P Component | Canonical Coefficient |
| --- | --- | --- | --- |
| 1. Work Environment | *a1* | 3 | .051 |
|  |  | 4 | .128 |
|  |  | 9 | .013 |
|  |  | 10 | .208 |
|  |  | 11 | .033 |
|  |  | 13 | .123 |
|  | *a2* | 1 | .187 |
|  |  | 2 | .026 |
|  |  | 5 | .077 |
|  |  | 6 | .118 |
|  |  | 7 | .019 |
|  |  | 8 | .042 |
|  |  | 12 | .067 |
| 2. Task | *b1* | 14 | .319 |
|  |  | 15 | .306 |
|  |  | 16 | .004 |
|  | *b2* | 17 | .153 |
|  |  | 18 | .088 |
|  |  | 19 | .254 |
| 3. Workload | *c1* | 20 | .002 |
|  |  | 22 | .002 |
|  |  | 23 | .007 |
|  |  | 26 | .589 |
|  |  | 27 | .087 |
|  | *c2* | 21 | .190 |
|  |  | 24 | .007 |
|  |  | 25 | .003 |
|  |  | 28 | .404 |
| 4. Schedule | *d1* | 29 | .005 |
|  |  | 31 | .003 |
|  |  | 33 | .002 |
|  |  | 36 | .871 |
|  | *d2* | 30 | .001 |
|  |  | 32 | .000 |
|  |  | 34 | .029 |
|  |  | 35 | .719 |
| 5. Function | *e1* | 39 | .735 |
|  |  | 42 | .010 |
|  |  | 43 | .009 |
|  |  | 44 | .009 |
|  |  | 46 | .002 |
|  | *e2* | 37 | .007 |
|  |  | 38 | .709 |
|  |  | 40 | .027 |
|  |  | 41 | .001 |
|  |  | 45 | .010 |
|  |  | 47 | .002 |
| 6. Role | *f1* | 48 | .302 |
|  |  | 50 | .341 |
|  | *f2* | 49 | .326 |
|  |  | 51 | .225 |
| 8. Autonomy | *h1* | 55 | .011 |
|  |  | 58 | .629 |
|  |  | 59 | .003 |
|  | *h2* | 56 | .014 |
|  |  | 57 | .620 |
| 10. Home/Work Interface | *j1* | 63 | .004 |
|  |  | 66 | .642 |
|  | *j2* | 64 | .393 |
|  |  | 65 | .187 |

***Table S5.***

**Latent Correlations Among Work Content and Work Context Factors.**

|  | 1. | 2. | 3. | 4. | 5. | 6. | 7. | 8. | 9. | 10. |
| --- | --- | --- | --- | --- | --- | --- | --- | --- | --- | --- |
| 1. Work Environment | - | .212^***^ | .386^***^ | .347^***^ | -.078^*^ | .306^***^ | -.100^**^ | -.055 | .164^**^ | .008 |
| 2. Task | .036 | - | .815^***^ | .163^**^ | -.055 | .696^***^ | -.196^***^ | .305^***^ | .362^***^ | -.101 |
| 3. Workload | .254^***^ | .532^***^ | - | .447^***^ | -.207^***^ | .606^***^ | -.160^***^ | .183^**^ | .210^***^ | .055 |
| 4. Schedule | .286^***^ | .320^***^ | .137^***^ | - | -.248^***^ | .189^*^ | -.043 | .038 | -.003 | .101 |
| 5. Function | .048 | .163^***^ | -.028 | -.021 | - | .039 | .334^***^ | .327^***^ | .136^***^ | .107^*^ |
| 6. Role | .051 | .497^***^ | .142^*^ | -.015 | .211^***^ | - | .009 | .350^***^ | .474^***^ | .135 |
| 7. Career | -.018 | .128^**^ | -.011 | -.003 | .139^***^ | .160^***^ | - | .424^***^ | .073^*^ | .292^***^ |
| 8. Autonomy | .201^***^ | .227^***^ | -.021 | .020 | .251^***^ | .497^***^ | .276^***^ | - | .254^***^ | .458^***^ |
| 9. Relationships | .078^**^ | .198^***^ | .061^*^ | .036 | .137^***^ | .306^***^ | .090^***^ | .303^***^ | - | .011 |
| 10. Home/Work Interface | .446^***^ | .037 | .297^***^ | .379^***^ | .100^*^ | .045 | .117^**^ | .223^***^ | .135^**^ | - |

*Note*. Content factors are separated from work context factors within the table by a dotted line. Below the diagonal, correlations pertain to the group level (N=5301), while above the diagonal correlations pertain to the organizational level (N=1631). ^***^ *p*<.001; ^**^ *p*<.01; ^*^ *p*<.05.

**References**

Barrales‐Molina, V., Bustinza, Ó. F., & Gutiérrez‐Gutiérrez, L. J. (2013). Explaining the causes and effects of dynamic capabilities generation: A multiple‐indicator multiple‐cause modelling approach. *British Journal of Management, 24*(4), 571-591. DOI:10.1111/j.1467-8551.2012.00829.

Bollen, K., & Lennox, R. (1991). Conventional wisdom on measurement: A structural equation perspective. *Psychological Bulletin,* *110*(2), 305-314. DOI:10.1037/0033-2909.110.2.305.

Bollen, K.A., & Bauldry, S. (2011). Three Cs in measurement models: Causal indicators, composite indicators, and covariates. *Psychological Methods, 16*(3), 265-284. DOI:10.1037/a0024448.

Bollen, K.A., & Diamantopoulos, A. (2017). In defense of causal-formative indicators: A minority report. *Psychological Methods, 22*(3), 581-596. DOI:10.1037/met0000056.

Costa, A.C., & Anderson, N. (2011). Measuring trust in teams: Development and validation of a multifaceted measure of formative and reflective indicators of team trust. *European Journal of Work and Organizational Psychology*, *20*(1), 119-154. DOI:10.1080/13594320903272083.

Costa, D.S.J. (2015). Reflective, causal, and composite indicators of quality of life: A conceptual or an empirical distinction? Qual Life Res, 24, 2057–2065. DOI 10.1007/s11136-015-0954-2.

Howell, R.D., Breivik, E., & Wilcox, J.B. (2007). Reconsidering formative measurement. *Psychological Methods, 12*(2), 205-218. DOI:10.1037/1082-989X.12.2.205

Inail (2014). *Indagine nazionale sulla salute e sicurezza sul Lavoro*. Milano: Inail.

Jarvis, C.B., MacKenzie, S.B., & Podsakoff, P.M. (2003). A critical review of construct indicators and measurement model misspecification in marketing and consumer research. *Journal of Consumer Research, 30*(2), 199-218. DOI:10.1086/376806

Kim, G., Shin, B., & Grover, V. (2010). Investigating two contradictory views of formative measurement in information systems research*. Management Information Systems Quarterly, 34*, 345-365.

MacKenzie, S.B., Podsakoff, P.M., & Jarvis, C.B. (2005). The Problem of Measurement Model Misspecification in Behavioral and Organizational Research and Some Recommended Solutions. *Journal of Applied Psychology, 90*(4), 710-730. DOI:10.1037/0021-9010.90.4.710.

Treiblmaier, H., Bentler, P.M., & Mair, P. (2011). Formative constructs implemented via common factors. *Structural Equation Modeling, 18*(1), 1-17. DOI:10.1080/10705511.2011.532693.

Willoughby, M.T., Kuhn, L.J., Blair, C.B., Samek, A., & List, J.A. (2017). The test–retest reliability of the latent construct of executive function depends on whether tasks are represented as formative or reflective indicators. *Child Neuropsychology, 23*(7), 822-837. DOI: 10.1080/09297049.2016.1205009.
